# The complete chloroplast genome of *Lonicera pampaninii* Levl. and its phylogenetic analysis

**DOI:** 10.1080/23802359.2021.1978891

**Published:** 2021-09-22

**Authors:** Chunyan Jiang, Shaoxiong Wu, Xiayu Feng, Chenju Yang, Zhengwen Yu

**Affiliations:** School of Life Sciences, Guizhou Normal University, Guiyang, China

**Keywords:** *Lonicera pampaninii*, Caprifoliaceae, complete chloroplast genome, phylogenetic

## Abstract

*Lonicera pampaninii* Levl, a Chinese herbal medicine widely used in the folk, has the effect of clearing away heat and detoxifying similar to other plants of the *Lonicera*. However, its genetic relationship with these plants is unclear. In this work, the cp genome of *Lonicera pampaninii* Levl. was assembled by the high-throughput Illumina pair-end sequencing data. The circular cp genome is 155,249 bp in size, including a large single-copy (LSC) region of 89,068 bp and a small single-copy (SSC) region of 18,635 bp, which were separated by two inverted repeat (IR) regions (23,773 bp each). A total of 120 genes were predicted, including eight ribosomal RNAs (rRNAs), 33 transfer RNAs (tRNAs), and 79 protein-coding genes (PCGs). Furthermore, phylogenetic analysis revealed a strong sister relationship between *L. pampaninii* and other two congeneric species (*Lonicera confusa* and *Lonicera japonica*). This study provides useful information for future genetic study of *L. pampaninii*.

*Lonicera pampaninii* Levl. is commonly known as ‘chicken bone tree’, belongs to the family Caprifoliaceae. It has the effects of expelling wind, clearing up heat and detoxification and commonly used to treat colds, coughs, sore throat, red eyes, swelling and pain, lung carbuncle, breast carbuncle, wet sores. *L. pampaninii* is mostly distributed in North China, Shaanxi, Gansu, Hubei, Sichuan, Yunnan, Guizhou and other regions with large reserves (Wei et al. 1999). In addition, the stems of *L. pampaninii* can also be used as a material to make artificial cotton. The soil of seed from *L. pampaninii* can make soap, and its vine can be used for garden greening (Duan et al. 1986; Wei et al. 1999). It is reported that luteolin, inositol, saponins, tannins, chlorogenic acid, isochlorogenic acid and other chemical components have been isolated from *Lonicera japonica*, and the content of chlorogenic acid has been determined (Peng et al. 2000; Xu et al. 2007; Lin et al. 2008; Zheng et al. 2012; Xiong et al. 2013; Yu et al. 2015; Kong et al. 2017). The chemical composition of *L. pampaninii* is similar to *Lonicera japonica* according to previous reports (Duan et al. 1986). Moreover, most researches show that caffeoylquinic acid, cerebrosides, nitrogen-containing iridoid glycosides and triterpene glycosides from *Lonicera japonica* have anti-allergic, anti-inflammatory, antibacterial and anti-bacterial antiviral activities. Although *L. pampaninii* has high economic value, few studies have focused on genetics of this plant for its distribution area and hidden growth environment. In this work, the complete chloroplast (cp) genome of *L. pampaninii* has been assembled and determined. The study provides useful information for future genetic research of *L. pampaninii*.

Young and healthy leaf samples were collected from Dangwu Town, Huaxi District, Guiyang City, Guizhou province, China (26.39005°N, 106.612275°E, 1197.5 m above sea level). The plants were identified using a species identification key by Dr. Chunyan Han, Kunming Caizhi Biotechnology Co. Ltd, Kunming, Yunnan, China. The leaf specimen (accession number: GZNUYZW202101001) was deposited in the herbarium of School of Life Sciences, Guizhou Normal University. The total genomic DNA (No. YX20210115901) was extracted using E.Z.N.A Plant DNA kit (FEIYANG, Guangzhou, China) and stored at −80 °C in the laboratory (room number: 1403) of School of Life Sciences, Guizhou Normal University. The genomic DNA of the sample is tested, and the DNA is interrupted by physical methods (ultrasound) after passing the test. The interrupted DNA is purified to construct a sequence library.First, extract the phyllochondrial genomic DNA, then use 1% agarose gel electrophoresis to detect and collect the phyllochondrial genomic DNA, and finally build a library with an initial amount of 1000 mg DNA.Break the total DNA into 400 bp fragments, filter reads with low sequencing quality, andfilter duplication reads to obtain high-quality clean data. Genomic DNA was extracted from fresh leaves and then sequenced using the Illumina NovaSeq platform (Illumina, San Diego, CA). The filtered reads were assembled using the program GetOrganelle (Jin et al. 2020) with *Lonicera japonica* (GenBank accession number: MH028738) as the initial reference genome. The assembled cp genome was annotated using the online software GeSeq (Tillich et al. 2017) by comparing the sequences with the cp genome of *Lonicera japonica*. The accurate annotated complete cp genome was submitted to GenBank with accession number MZ241298.

The length of the complete cp genome sequence of *L. pampaninii* is 155,249 bp, consisting of a large single-copy (LSC, 89,068 bp) region, a small single-copy (SSC, 18,635 bp) region, and two inverted repeat (IRA and IRB) regions of 23,773 bp respectively. Totally, 120 genes were predicted, including 79 protein-coding genes (PCGs), eight ribosomal RNA (rRNA) genes, and 33 transfer RNA (tRNA) genes. Among these assembled genes, all rRNAs, three PCGs (rps7, rps12, ndhB, ycf2, and ycf15) and seven tRNAs (trnH-GUG, trnL-CAA, trnV-GAC, trnL-GAU, trnA-UGC, trnR-ACG, and trnN-GUU) were with double copies. Intron-exon analysis showed the majority (103 genes, 86%) genes with no introns, whereas 17 genes (14%) contain introns.

To further understand the cp genome of *L. pampaninii*, 21 cp genome sequences of Caprifoliaceae family (one *Triosteum* species, 13 *Lonicera* species, one *Weigela* species, two species from *Dipelta* genus, one *Heptacodium* species, and three species from *Patrinia* genus,) were downloaded from GenBank to construct the phylogenetic trees through maximum-likelihood (ML) analysis. The ML tree based on GTR + gamma + I model was performed using RAxML (Version 8.0.19) with 1000 bootstrap replicates (Stamatakis 2014). The phylogenetic tree indicated that *L. pampaninii* belongs to *Lonicera* genus (Figure 1), and showed a strong sister relationship between *L. pampaninii* and other two congeneric species (*Lonicera confusa* and *Lonicera japonica*).

**Figure 1.**
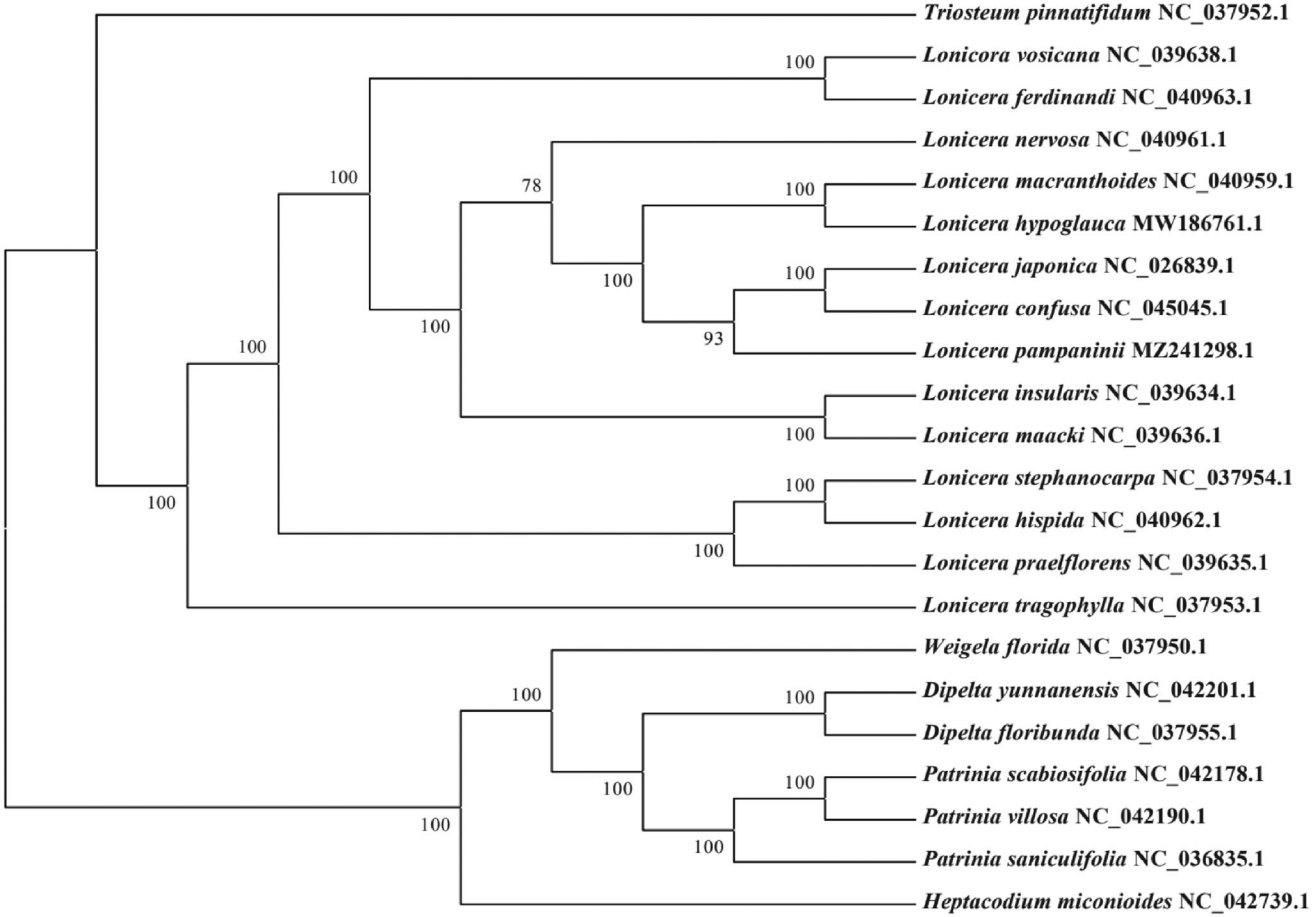
Maximum-likelihood tree based on the complete cp genome sequences of 21 species from the Caprifoliaceae. GenBank accession numbers are described in the figure. Shown next to the nodes are bootstrap support values based on 1000 replicates.

## Data Availability

The genome sequence data that support the findings of this study are openly available in GenBank of NCBI at https://www.ncbi.nlm.nih.gov, reference number MZ241298. The associated BioProject, SRA, and Bio-Sample numbers are PRJAN752821, SUB10196548, and SAMN20608006 respectively.
